# Marked progress in AL amyloidosis survival: a 40-year longitudinal natural history study

**DOI:** 10.1038/s41408-021-00529-w

**Published:** 2021-08-04

**Authors:** Andrew Staron, Luke Zheng, Gheorghe Doros, Lawreen H. Connors, Lisa M. Mendelson, Tracy Joshi, Vaishali Sanchorawala

**Affiliations:** 1 Amyloidosis Center, Boston University School of Medicine and Boston Medical Center, Boston, MA USA; 2 Section of Hematology and Oncology, Boston University School of Medicine and Boston Medical Center, Boston, MA USA; 3 Department of Biostatistics, Boston University School of Public Health, Boston, MA USA; 4 Department of Pathology and Laboratory Medicine, Boston University School of Medicine and Boston Medical Center, Boston, MA USA

**Keywords:** Haematological diseases, Myeloma

## Abstract

The recent decades have ushered in considerable advancements in the diagnosis and treatment of systemic light chain (AL) amyloidosis. As disease outcomes improve, AL amyloidosis-unrelated factors may impact mortality. In this study, we evaluated survival trends and primary causes of death among 2337 individuals with AL amyloidosis referred to the Boston University Amyloidosis Center. Outcomes were analyzed according to date of diagnosis: 1980-1989 (era 1), 1990-1999 (era 2), 2000-2009 (era 3), and 2010-2019 (era 4). Overall survival increased steadily with median values of 1.4, 2.6, 3.3, and 4.6 years for eras 1–4, respectively (*P* < 0.001). Six-month mortality decreased over time from 23% to 13%. Wide gaps in survival persisted amid patient subgroups; those with age at diagnosis ≥70 years had marginal improvements over time. Most deaths were attributable to disease-related factors, with cardiac failure (32%) and sudden unexpected death (23%) being the leading causes. AL amyloidosis-unrelated mortality increased across eras (from 3% to 16% of deaths) and with longer-term survival (29% of deaths occurring >10 years after diagnosis). Under changing standards of care, survival improved and early mortality declined over the last 40 years. These findings support a more optimistic outlook for patients with AL amyloidosis.

## Introduction

Systemic light chain (AL) amyloidosis was historically regarded as an inevitably fatal disease. Prior to effective treatments against the underlying plasma cell dyscrasia, prognosis was dismal with an expected survival of 13 months and, among those with symptomatic heart involvement, only 6 months [1–3]. A turning point came in the 1990s with the introduction of high-dose melphalan and autologous stem cell transplantation (HDM/SCT) for AL amyloidosis [4, 5]. This treatment modality offered a more favorable prognosis for carefully selected patients [6]. However, options remained limited for 65–75% of newly diagnosed patients who were ineligible for HDM/SCT. A wave of new therapeutics for AL amyloidosis arrived after 2010, including bortezomib-based regimens, novel proteasome inhibitors (PI), next-generation immunomodulatory drugs (IMiD), and anti-CD38 monoclonal antibodies [7–13]. Beyond treatments, standardized risk-stratification and treatment response assessment, owing to the advent of the serum-free light-chain assay and organ dysfunction biomarkers in the 2000s, contributed to a much-improved outlook for this rare disease [14–19].

The magnitude of impact on survival from these advancements is partially defined by the observed increase in disease prevalence of 12% per year, despite an unchanging incidence rate [20, 21]. At major referral centers for AL amyloidosis, survival rates have improved over the years [22–24]. Long-term survival is becoming more common with upwards of 1 in 5 patients now attaining longevity of 10 years after diagnosis [25]. Even longer-term survival of 15–20 years is seen in ~30% of patients treated with HDM/SCT [6, 26, 27]. As survival improves, there may be a shift in primary causes of death among patients with AL amyloidosis. Leading causes during early course of disease include progressive heart failure, arrhythmias, and sudden unexpected death [1, 28, 29]. Causes of death later in the course of disease are less clear. The long-term effects on mortality of AL amyloidosis and its treatments, along with co-morbid conditions, have yet to be clarified in a broad patient population.

Here, we illustrate the progress made in the survival of patients with AL amyloidosis. Unlike prior reports, we focus on the distribution of survival improvements across various patient subgroups, in order to identify those with the highest unmet needs. We also examine primary causes of death and their relation to AL amyloidosis to better inform disease biology and natural history over time.

## Methods

### Data source and study population

Patients with AL amyloidosis diagnosed between January 1980 and December 2019 were identified from the prospectively maintained database at the Amyloidosis Center at Boston University School of Medicine and Boston Medical Center. Those with localized AL amyloidosis, myeloma-associated AL amyloidosis, and B cell lymphoproliferative disorder-associated AL amyloidosis were excluded from analysis due to differing risks and treatment approaches [30–35]. When necessary, typing of amyloidogenic protein was performed by immunohistochemistry, immunogold electron microscopy (IG-EM) or liquid chromatography and tandem mass spectrometry (LCMS^2^). Patients with indeterminate amyloid type were excluded. A total of 2337 patients met criteria for the diagnosis of systemic AL amyloidosis alone, with positive Congo red staining of biopsy specimen and plasma cell clonality in the context of appropriate clinical syndrome. All patients provided written consent for research under the approval of the Institutional Review Board and in accordance with the Declaration of Helsinki (ClinicalTrials.gov Identifier: NCT00898235).

Individuals were categorized into 4 strata according to the date of tissue diagnosis, each at 10-year intervals: 1980–1989 (era 1), 1990–1999 (era 2), 2000–2009 (era 3), and 2010–2019 (era 4). Descriptive data included demographics, hematologic parameters, organ system involvement and treatments. After 2005, organ involvement was defined according to the consensus criteria of the International Society of Amyloidosis (ISA) [36]. Prior to this, it was determined by experts at the institution using available clinical information. When assessing organ involvement, only the heart, kidney, liver, and nervous system were included. Cardiac biomarker stage was assigned based on predefined criteria [16] for individuals diagnosed after 2007, when B-type natriuretic peptide (BNP, normal <53.2 pg/mL) and troponin-I (normal <0.013 ng/mL) were both available.

For treatment analysis, all plasma cell-directed therapies received throughout the course of disease were counted. Non-plasma cell-directed therapies were excluded (e.g., doxycycline, *n* = 14; NEOD001, *n* = 10). Treatment data were confirmed for 1581 patients. The remaining patients did not receive therapy due to early death (*n* = 253) and other reasons (e.g., absence of vital organ involvement, poor performance status or patient choice, *n* = 82), or treatment could not be confirmed due to lack of follow-up (*n* = 421). Therapies were classified as: HDM/SCT; oral melphalan-based regimen; PI-based regimen; IMiD-based regimen; anti-CD38 monoclonal antibody; or other treatment (e.g., anthracycline with dexamethasone, dexamethasone monotherapy).

### Survival and cause of death ascertainment

Vital status was verified by yearly clinical evaluations. Contact by letter and/or phone was established with patients who did not return for follow-up. If contact could not be established, survival information was ascertained from the U.S. Social Security Death Index. Data cutoff was October 2020. For deceased patients, the cause of death and its relation to AL amyloidosis was determined by treating physicians using information from medical documentation, referring physicians, family members and/or official documents of death. Sufficient data were accessible on 1243 (75%) of 1660 deaths to confidently assign as related or unrelated to AL amyloidosis. Deaths were defined as AL amyloidosis-related when clinical information indicated progression of amyloid organ disease or complications from plasma cell-directed therapy. Deaths occurring while in remission, off treatment and without evidence of amyloid organ disease progression, or with clear relation to a co-morbid condition were considered AL amyloidosis-unrelated. The primary cause of death was available for 1160 (70%) cases and categorized as: organ failure; sudden unexpected death (e.g., occurring during sleep, or shortly after symptom onset in patients whose disease was not severe enough to explain rapid clinical deterioration); infection and/or sepsis; treatment-related complication; major vascular event (e.g., stroke, myocardial infarction, pulmonary embolism or other thromboembolic event); hemorrhage; malignancy; or other cause (e.g., trauma/suicide, cachexia, bowel obstruction, dementia).

### Statistical analysis

Data were presented in chronological order, unless stated otherwise. For descriptive analyses, chi-square and one-way ANOVA tests were used to make comparisons between eras. If data were missing, the number of complete cases (*N*) were specified. There were more incomplete data during the earlier eras, largely due to variables not yet being included in the database or the unavailability of biomarker tests. Overall survival (OS) was measured from date of diagnosis to death (any cause) or last follow-up (censored), and illustrated using Kaplan–Meier method with differences between eras compared using the log-rank test. Survival probabilities and 95% confidence intervals (CI) were calculated. Early mortality was defined as deaths occurring within 6 months of diagnosis. Statistical computations were performed by SAS version 9.4 and R 4.0.2 software with *P* < 0.05 set as statistically significant.

## Results

The number of individuals in each stratum was: era 1, *n* = 185 (8% of all patients); era 2, *n* = 575 (25%); era 3, *n* = 865 (37%); and era 4, *n* = 712 (30%). Median time to evaluation at the Amyloidosis Center from diagnosis was 2 months (interquartile range, 1–5 months). Patient and disease characteristics at initial presentation to the referral center are shown in Table 1. Over the 40-year period, median age at diagnosis increased by 4 years. The proportion of patients with age ≥75 years increased threefold. There was a downward trend in the difference between involved and uninvolved free light chains (dFLC). This was paralleled by a shortening time interval to diagnosis from patient-reported symptom onset, from 10 months in era 1 to 6 months in era 4 (*P* = 0.065). Differences in organ system involvement and biomarkers had no apparent pattern by chronological era.

**Table 1:** Patient characteristics and treatments by era of diagnosis.

	All eras (1980–2019)	Era 1 (1980-1989)	Era 2 (1990-1999)	Era 3 (2000-2009)	Era 4 (2010–2019)	*P* value
*N*	2337	185	575	865	712	–
Median age, years (IQR)	61 (53–68)	59 (52–66)	60 (52–68)	60 (53–68)	63 (56–69)	<0.001
Age ≥75 years, *n* (%)	209 (9)	7 (4)	37 (6)	80 (9)	85 (12)	<0.001
Male, *n* (%)	1405 (60)	111 (60)	344 (60)	530 (61)	420 (59)	0.830
Racial/ethnic minority, *n* (%)	312 (13)	11 (6)	43 (7)	112 (13)	146 (21)	<0.001
Time to diagnosis from symptom onset (*N* = 2095), months (IQR)	7 (2–13)	10 (4–17)	7 (3–14)	7 (3–13)	6 (1–12)	0.065
Hematologic parameters						
Amyloidogenic LC, *n* (%)						0.212
Lambda (%)	1673 (79)	57 (70)	396 (78)	667 (78)	570 (80)	–
Kappa (%)	458 (21)	24 (30)	110 (22)	192 (22)	142 (20)	–
Median dFLC (*N* = 1457), mg/L (IQR)^a^	90 (27–252)	–	136 (45–285)	98 (30–276)	76 (23–219)	0.229
Organ involvement, *n* (%)						
Cardiac (*N* = 1859) (%)	1176 (63)	87 (67)	171 (53)	487 (70)	431 (61)	<0.001
Renal (*N* = 2017) (%)	1579 (78)	92 (72)	362 (88)	637 (82)	488 (70)	<0.001
Hepatic (*N* = 1742) (%)	491 (28)	35 (31)	147 (48)	219 (36)	90 (13)	<0.001
Nervous system (*N* = 1712) (%)	656 (38)	56 (46)	116 (42)	259 (43)	225 (32)	<0.001
≥2 organs (%)	1187 (53)	88 (62)	193 (37)	517 (60)	389 (55)	<0.001
Organ biomarkers, median (IQR)^b^						
BNP (*N* = 1159), pg/mL	202 (65–574)	–	–	187 (57–486)	216 (73–630)	0.038
Troponin-I (*N* = 999), ng/mL	0.05 (0.01–0.13)	–	–	0.05 (0.02–0.15)	0.04 (0.01–0.13)	0.157
Proteinuria (*N* = 2089), g per 24 h	1.6 (0.1–6.4)	1.1 (0–5.0)	2.0 (0.2–6.6)	1.0 (0.1–5.7)	2.5 (0.2–7.1)	0.479
eGFR (*N* = 1930), mL/min/1.73 m^2^	68 (38–92)	65 (46–82)	63 (33–92)	68 (38–92)	70 (40–92)	0.198
ALP (*N* = 1914), IU/L	94 (71–141)	84 (65–142)	101 (78–166)	95 (72–142)	90 (69–130)	<0.001
BNP-based cardiac stage, *n* (%)						
Stage I (%)	279 (28)	–	–	92 (31)	187 (27)	0.126
Stage II (%)	426 (43)	–	–	117 (40)	309 (44)	0.240
Stage IIIa (%)	139 (14)	–	–	51 (17)	88 (12)	0.043
Stage IIIb (%)	155 (16)	–	–	34 (12)	121 (17)	0.026
Receipt of PC-directed therapy						
Treatment confirmed, *n*^c^	1581	57	395	598	531	–
≥2 treatments, *n* (%)	594 (25)	0 (0)	127 (22)	232 (27)	231 (32)	<0.001
Treatment classifications (any line), *n* (%)						
HDM/SCT (%)	723 (31)	–	229 (40)	320 (37)	174 (24)	<0.001
PI-based (%)	580 (25)	–	16 (3)	143 (17)	421 (59)	<0.001
Melphalan-based (%)	570 (24)	57 (31)	243 (42)	241 (28)	29 (4)	<0.001
IMiD-based (%)	362 (15)	–	41 (7)	169 (20)	151 (21)	<0.001
Anti-CD38 monoclonal antibody (%)	92 (4)	–	1 (<1)	14 (2)	77 (11)	<0.001
Other (%)	62 (3)	–	28 (5)	33 (4)	1 (<1)	<0.001

^a^Values were measured at the time of initial evaluation at the referral center and some may have instituted treatment in the community before initial evaluation. The serum free light chain assay was unavailable prior to 2003. Testing was performed retrospectively on frozen sera of 96 patients from era 2.

^b^BNP and troponin-I were introduced in the mid-2000s.

^c^The remaining patients did not receive therapy, or receipt of treatment could not be confirmed.

*IQR* interquartile range, *LC* light chain, *dFLC* difference between involved and uninvolved free light chains, *BNP* brain natriuretic peptide, *eGFR* estimated glomerular filtration rate by CKD-EPI equation, *ALP* alkaline phosphatase, *PC* plasma cell, *HDM/SCT* high-dose melphalan and autologous stem cell transplantation, *PI* proteasome inhibitor, *IMiD* immunomodulatory drug.

The assortment of plasma cell-directed treatments (including first and subsequent lines) received by individuals from each stratum are presented in Table 1. Oral melphalan-based regimens were the sole option during era 1. Some individuals from eras 2 and 3 survived long enough to receive the novel agents introduced after 2010. HDM/SCT was the most common treatment, used for 31% (*n* = 723) of patients. Use of HDM/SCT declined from eras 2 to 4. Meanwhile, PI-based regimens emerged as the more common treatment, used for 59% (*n* = 421) of patients in the latest era.

### Survival and early mortality outcomes

**Fig. 1:**
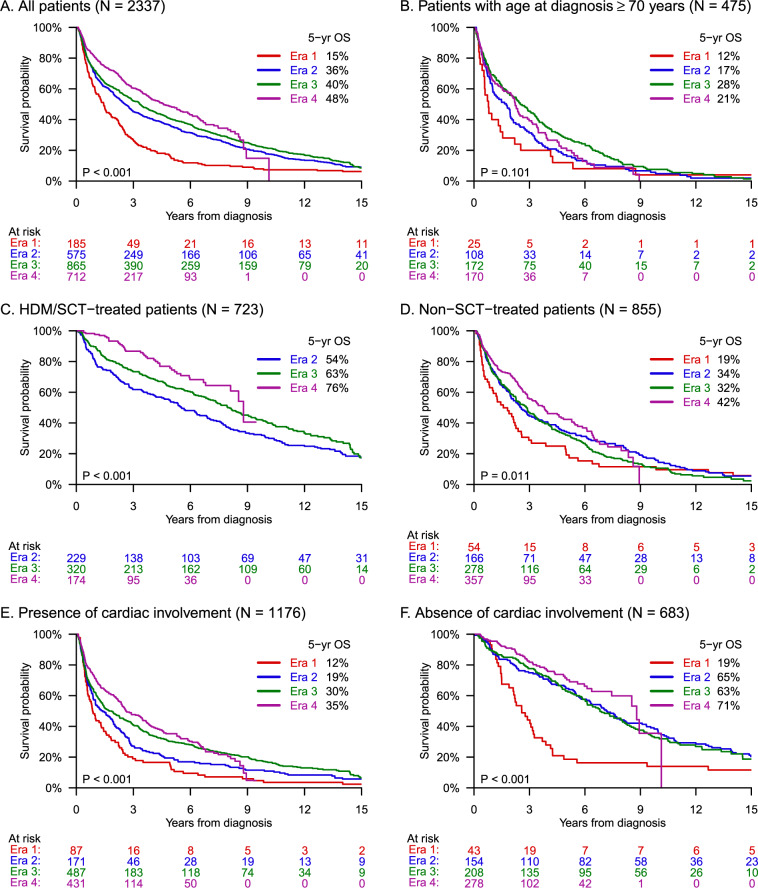
Trends in overall survival over a 40-year period among patients with AL amyloidosis. Kaplan–Meier survival curves by era of diagnosis for the entire study population (**A**) and patient subgroups (**B**–**F**).

At data cutoff, a total of 1660 (71%) individuals had died from any cause, including 182 (98%), 532 (93%), 663 (77%), and 283 (40%) individuals from eras 1 to 4, respectively. Kaplan–Meier curves for the entire cohort are shown in Fig. 1A. Median OS increased significantly (*P* < 0.001) with a value of 4.6 years (95% CI, 3.8–5.6 years) for individuals diagnosed during the latest era, compared to 1.4 years (95% CI, 1.0–8 years) for those diagnosed during the first era (Table 2). Survival improved most drastically between eras 1 and 2, whereas it nearly plateaued between eras 2 and 3. The 5-year OS rate increased with each era from 15% to 36% to 40% to 48%, and the 10-year OS rate from 7% to 18% to 22% (non-evaluable for era 4). The 20-year OS rate for the entire cohort was 4% (95% CI, 3–5%). In a multivariate analysis (Table 3), hazard ratios for mortality were even more drastic across eras after adjusting for baseline characteristics.

**Table 2:** Survival intervals and rates by era of diagnosis.

	All eras (1980–2019)	Era 1 (1980–1989)	Era 2 (1990–1999)	Era 3 (2000–2009)	Era 4 (2010–2019)
All patients					
Deaths, *n* (%)	1660 (71)	182 (98)	532 (93)	663 (77)	283 (40)
Median age at death, years (IQR)	66 (58–73)	63 (56–69)	66 (57–73)	66 (58–73)	68 (61–75)
Median OS, years (95% CI)	3.0 (2.8–3.4)	1.4 (1.0–1.8)	2.6 (2.2–3.0)	3.3 (2.9–3.8)	4.6 (3.8–5.6)
6-month mortality rate, % (95% CI)	16 (15–18)	23 (18–30)	17 (14–21)	17 (14–19)	13 (10–16)
2-year OS, % (95% CI)	60 (58–62)	41 (34–48)	56 (52–60)	60 (57–64)	71 (67–75)
10-year OS, % (95% CI)	20 (18–22)	7 (4–12)	18 (15–21)	22 (19–25)	–
Subgroups					
Median OS, years (95% CI)					
Age ≥70 years	2.1 (1.8–2.4)	0.8 (0.5–1.6)	1.8 (1.0–2.0)	2.5 (1.8–3.3)	2.2 (1.6–2.6)
HDM/SCT-treated	7.6 (6.8–8.2)	–	5.5 (4.5–6.9)	8.0 (6.9–9.2)	8.8 (8.1–NR)
Non-SCT-treated	2.9 (2.6–3.3)	1.6 (0.8–2.3)	2.6 (2.0–3.5)	2.9 (2.3–3.3)	3.8 (2.9–4.8)
Presence of cardiac involvement	2.0 (1.6–2.3)	0.9 (0.6–1.3)	1.4 (0.9–2.0)	1.7 (1.4–2.3)	2.6 (2.4–3.5)
Absence of cardiac involvement	7.0 (6.1–7.9)	2.6 (1.9–3.2)	7.2 (5.8–8.3)	6.8 (5.9–8.2)	8.8 (7.5–10.1)

*IQR* interquartile range, *OS* overall survival, *CI* confidence interval, *NR* not reached.

**Table 3:** Univariate and multivariate analyses of mortality risk.

	Univariate		Multivariate	
	HR (95% CI)	*P* value	HR (95% CI)	*P* value
Era 1 (1980–1989)	2.06 (1.70–2.49)	<0.001	2.70 (2.04–3.57)	<0.001
Era 2 (1990–1999)	1.41 (1.22–1.63)	<0.001	1.47 (1.20–1.79)	<0.001
Era 3 (2000–2009)	1.29 (1.12–1.48)	<0.001	1.29 (1.10–1.52)	0.002
Era 4 (2010–2019)	Reference		Reference	
Age at diagnosis	1.02 (1.02–1.03)	<0.001	1.03 (1.03–1.04)	<0.001
Racial/ethnic minority	0.98 (0.84–1.14)	0.771	1.19 (0.99–1.42)	0.065
Time to diagnosis from symptom onset	1.01 (1.00–1.01)	<0.001	1.00 (1.00–1.01)	0.200
Heart involvement	2.27 (2.00–2.57)	<0.001	2.33 (2.03–2.69)	<0.001
Kidney involvement	0.98 (0.86–1.12)	0.730	1.03 (0.89–1.19)	0.719

*HR* hazard ratio, *CI* confidence interval.

Mortality within 6 months of diagnosis decreased over time from 23% (95% CI, 18–30%) in era 1 to13% (95% CI, 10–16%) in era 4. Individuals from the latest era who experienced early mortality (Table 4) were older in age (median, 68 years vs. 62 years) and more likely to be from an underrepresented racial/ethnic group (27% vs. 20%) as compared to their surviving counterparts. They also had a higher median dFLC (267 mg/L) at baseline, predominance of cardiac involvement (92%) and more advanced cardiac stage (50% with stage IIIb disease).

**Table 4:** Characteristics of patients from the latest era who experienced early mortality.

	2010–2019
*N*	88
Median age, years (IQR)	68 (59–74)
Age ≥75 years, *n* (%)	20 (23%)
Male, *n* (%)	51 (58%)
Racial/ethnic minority, *n* (%)	24 (27%)
Time to diagnosis from symptom onset, months (IQR)	8 (4–16)
Hematologic parameters	
Amyloidogenic LC, *n* (%)	
Lambda (%)	66 (75)
Kappa (%)	22 (25)
Median dFLC, mg/L (IQR)	267 (108–489)
Organ involvement, *n* (%)	
Cardiac (%)	81 (92)
Renal (%)	58 (67)
Hepatic (%)	23 (26)
Nervous system (%)	40 (46)
≥2 organs (%)	68 (77)
Organ biomarkers, median (IQR)	
BNP (*N* = 86), pg/mL	916 (393–1783)
Troponin-I, ng/mL	0.16 (0.07–0.50)
Proteinuria (*N* = 76), g per 24 h	1.3 (0.3–5.6)
eGFR (*N* = 86), mL/min per 1.73 m^2^	52 (31–83)
ALP, IU/L	124 (78–203)
BNP-based cardiac stage (*N* = 86), *n* (%)	
I (%)	2 (2)
II (%)	30 (35)
III (%)	11 (13)
IIIb (%)	43 (50)

*IQR* interquartile range, *LC* light chain, *dFLC* difference between involved and uninvolved free light chains, *BNP* brain natriuretic peptide, *eGFR* estimated glomerular filtration rate by CKD-EPI equation, *ALP* alkaline phosphatase.

### Patients aged ≥70 years

While median OS improved among patients with age at diagnosis < 70 years from 1.5 years (95% CI, 1.2–2.1 years) to 6.6 years (95% CI, 5.5–7.5 years), the improvement was marginal and statistically non-significant among patients with age ≥70 years. Median OS in this older subgroup (*N* = 475, Fig. 1B) increased from 0.8 years (95% CI, 0.5–1.6 years) in the first era to 2.2 years (95% CI, 1.6–2.6 years) in the last era (*P* = 0.101). Also, 6-month mortality was continually high across eras for older patients (23% in era 4).

### HDM/SCT-treated patients

Among 1581 patients with confirmed treatment, median OS was 7.6 years (95% CI, 6.8–8.2 years) for those who underwent HDM/SCT either upfront or after induction (*N* = 726, Fig. 1C) and 2.9 years (95% CI, 2.6–3.3 years) for those who received only non-SCT treatments (*N* = 855, Fig. 1D). Survival improved significantly over time for both subgroups, but HDM/SCT-treated patients had particularly large gains. During eras 2–4, their 5-year OS rate improved from 54% to 63% to 76%, compared to 34% to 32% to 42% for individuals treated with non-SCT therapies. Early mortality among HDM/SCT-treated patients decreased from 10% to 6% to 2%, whereas for non-SCT-treated patients it decreased between eras 1 and 2 from 26% to 11%, then remained fixed at 11% thereafter. Notably, 397 (55%) patients in the HDM/SCT-treated subgroup and 197 (23%) patients in the non-SCT subgroup received ≥2 lines of treatment. Stage ≥III cardiac involvement was present in 16% (46/287) and 35% (165/473) of those treated with HDM/SCT and non-SCT therapies, respectively.

### Cardiac involvement

A total of 1859 patients were evaluable for cardiac involvement based on predefined criteria; the remainder were equivocal. Among individuals with presence of cardiac involvement (*N* = 1176, Fig. 1E), median OS improved between eras 1 and 4 from 0.9 years (95% CI, 0.6–1.3 years) to 2.6 years (95% CI, 2.4–3.5 years). The improvement was even more drastic for patients with absence of cardiac involvement (*N* = 683, Fig. 1F), increasing from 2.6 years (95% CI, 1.9–3.2 years) to 8.8 years (95% CI, 7.5–10.1 years). The 5-year OS rate for those with absence of cardiac involvement rose from 19% to 71%, with the majority of this gain (46 percentage points) occurring between eras 1 and 2. Additionally, this subgroup had a very low 6-month mortality rate of ≤5% across all eras. Meanwhile, early mortality was highest among patients with presence of cardiac involvement at 31% (95% CI, 13–42%) in era 1, but decreased to 19% (95% CI, 15–23%) in era 4.

**Fig. 2:**
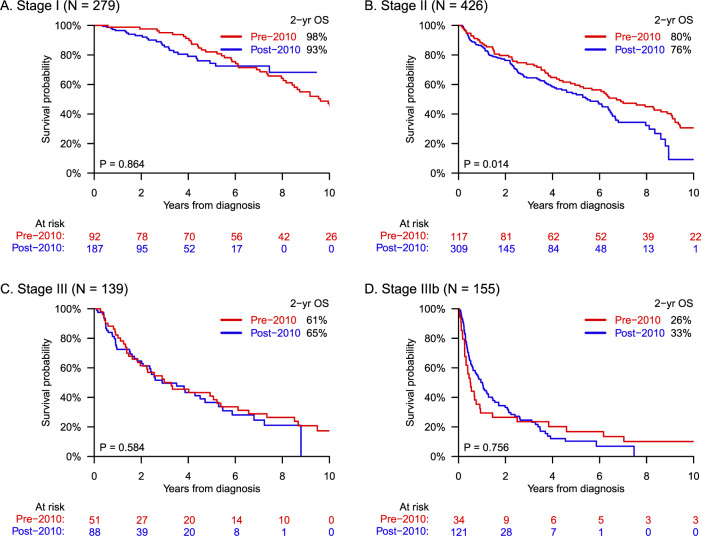
Survival according to BNP-based cardiac stage before and after 2010. Despite the introduction of bortezomib-based regimens in 2010, survival did not improve significantly for any cardiac stage I–IIIb, as shown in panels (**A**–**D**), respectively. Complete biomarker data sets (BNP and troponin-I) were not available until 2007. Accordingly, pre-2010 and post-2010 consisted of patients diagnosed in 2007–2009 and 2010–2019, respectively. The apparent decline in survival for cardiac stage II disease (**B**) may be the result of censorship bias due to shorter follow-up for the post-2010 group.

Among 999 patients with complete biomarker data sets, survival was analyzed according to cardiac stage (Fig. 2). There was no significant gain in survival for any BNP-based cardiac stage (I–IIIb) comparing individuals diagnosed before (*N* = 294) vs. after (*N* = 705) the year 2010, when bortezomib-based regimens were introduced into practice. For patients with cardiac stage IIIb disease, median OS was 0.5 years (95% CI, 0.2–0.9 years) and 1.0 years (95% CI, 0.6–1.3 years) before vs. after 2010, respectively; whereas 6-month mortality was 50% (95% CI, 35–68%) and 35% (95% CI, 27–45%). These differences were insignificant (*P* = 0.756).

### Cause of death

**Fig. 3:**
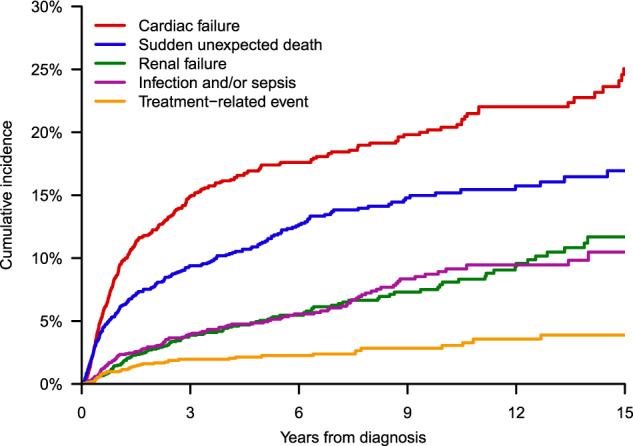
Cumulative incidences of the top causes of death across disease course. The rate of cardiac failure and sudden unexpected deaths decreased with longer survival time from diagnosis.

Primary cause of death was identified in 1160 cases (Table 5), of which 92 had confirmation by autopsy. Organ failure was the most common cause, accounting for 564 (49%) deaths, amongst which cardiac failure predominated. Sudden unexpected death was the next most frequent cause, contributing to 266 (23%) deaths. Cumulative incidences of the top causes of death over the course of disease are displayed in Fig. 3. Cardiac failure and sudden death decreased in proportion with longer survival from diagnosis, representing 67% (236/354) of deaths occurring within ≤ 6 months; 56% (322/575) within >6 months to ≤5 years; 36% (54/151) within >5 years to ≤10 years; and 36% (29/80) after >10 years (*P* < 0.001). Compared to early-occurring deaths, those at >5 years after diagnosis were more frequently caused by renal failure (18%), infections (16%), and secondary primary malignancies (7%). Solid tumors constituted most malignancies in this cohort: 17 of 21 cases. The remaining 4 cases were acute myeloid leukemias, thought to be therapy-related as these patients were previously treated with alkylating agents (2 with oral melphalan, 1 with oral melphalan followed by HDM/SCT, and 1 with cyclophosphamide).

**Table 5:** Primary causes of death in the AL amyloidosis cohort.

	All deaths	Early deaths (≤6 months)	Late deaths (>5 years)	Disease-related deaths	Disease-unrelated deaths^a^
Identified cause, *N*	1160	354	231	1042	103
Organ failure (%)	564 (49)	169 (48)	94 (41)	558 (54)	6 (6)
Cardiac (%)^b^	375 (32)	130 (37)	41 (18)	372 (36)	3 (3)
Renal (%)	124 (11)	13 (4)	41 (18)	121 (12)	3 (3)
Hepatic (%)	37 (3)	22 (6)	2 (1)	37 (4)	0 (0)
Autonomic (%)	28 (2)	4 (1)	10 (4)	28 (3)	0 (0)
Sudden unexpected death (%)^c^	266 (23)	106 (30)	42 (18)	246 (24)	5 (5)
Infection and/or sepsis (%)	125 (11)	25 (7)	36 (16)	95 (9)	30 (29)
Treatment-related event (%)	49 (4)	16 (5)	9 (4)	39 (4)	10 (10)
Major vascular event (stroke, MI, VTE) (%)	48 (4)	19 (5)	8 (3)	31 (3)	17 (17)
Hemorrhage (%)	33 (3)	12 (3)	10 (4)	29 (3)	4 (4)
Malignancy (%)	21 (2)	1 (<1)	16 (7)	4 (<1)	17 (17)
Other (%)	54 (5)	6 (2)	16 (7)	40 (4)	14 (14)
Unidentified cause, *N*	500	74	179	7	91

^a^Deaths occurring while in remission and off plasma cell-directed treatment, or with clear relation to a co-morbid condition. Relation to AL amyloidosis was evaluable for 98 cases despite an unidentified primary cause of death.

^b^Cardiac failure includes 36 (10%) cardiac arrhythmia events.

^c^There were 15 patients with sudden death for whom relation to AL amyloidosis was uncertain.

*MI* myocardial infarction, *VTE* venous thromboembolism.

### Disease-unrelated mortality

Of 1243 deaths with known relation to disease, the majority (91%, *n* = 1134) were AL amyloidosis-related. The proportion of deaths not attributable to AL amyloidosis or its treatment increased significantly (*P* < 0.001) with each era from 3% (4/133) to 8% (36/434) to 8% (38/483) to 16% (31/193). AL amyloidosis-unrelated deaths also increased with longer survival from diagnosis, accounting for 2% (9/373) of deaths occurring within ≤ 6 months; 8% (48/616) within >6 months to ≤5 years; 16% (25/161) within >5 years to ≤10 years; and 29% (27/93) after >10 years (*P* < 0.001). The most prevalent causes of AL amyloidosis-unrelated deaths were infections unrelated to treatment (29%), major vascular events (17%), and solid tumor malignancies (17%).

## Discussion

In this longitudinal natural history study, we collected and analyzed real-world data on survival among patients with AL amyloidosis seen at a major U.S. referral center. Median OS improved from 1.4 years in the 1980s to 4.6 years in the 2010s. This finding is in line with a study of patients with AL amyloidosis from the U.K., which reported an increase in survival from 17 months (1.4 years) in 1987–2005 to 51 months (4.25 years) in 2011–2015 [24]. The observed 5-year OS rate of 48% (95% CI, 43–53%) in the latest era of our study, however, falls short of the relative 5-year OS rate of 55.6% (95% CI, 54.9–56.3%) that was estimated for multiple myeloma in 2011–2017 [37], supporting the contention that these two diseases— although related—have divergent natural histories.

Additionally, we found that 6-month mortality decreased over our study period from 23% to 13%. This outcome contrasts earlier observations from both the Mayo Clinic and U.K. showing unchanging rates of early mortality. From 1977 to 2006, 43–46% of patients in the Mayo Clinic cohort were dying within one year of diagnosis [22]. In an update from the same center, 6-month mortality had abated for the first time after 2005, decreasing from a rate of 37% to 25% [23]. The lower early mortality rates observed in our study compared to other centers may be attributed to our institution being a major transplantation referral site, drawing younger and fitter patients with earlier-stage disease. One epidemiologic study suggested that the median age of patients with AL amyloidosis in the general population may, in fact, be over a decade older (i.e., 76 years) than that observed in our referred cohort [21]. Median age at diagnosis in our population was 61 years, whereas in the Mayo Clinic and U.K. cohorts was 63 and 66 years, respectively [22, 23]. Moreover, the distribution of cardiac stages I–III was 28%, 43% and 29% (BNP-based staging system) in our cohort, compared to 21%, 28% and 41% (Mayo 2004 staging system) in the Mayo Clinic cohort [23]. The survival trends portrayed in this study reflect the effects of institutional and community factors, in addition to global developments in communication, technology and medicine. Key predictors of survival in AL amyloidosis are the severity of cardiac involvement and the effectiveness of treatments to attain deep and durable hematologic responses [18]. We observed increasing receipt of ≥2 lines of therapy over time, owing to more available options for relapsed/refractory AL amyloidosis [38], which are adopted from the therapeutic landscape for multiple myeloma. Due to the pathological nature of AL amyloidosis, disease awareness and timely detection at the community level are also critical for survival outcomes. The pathway to diagnosis can be long and complicated. In an advocacy group-led questionnaire study, over one-third of patients with AL amyloidosis reported that their symptoms preceded diagnosis by more than one year [39]. Longer diagnostic interval was independently associated with higher risk of death in a recent report from our center [40]. However, findings from the current study provide grounds for optimism. The median time to diagnosis from symptom onset was shortened across the eras by 4 months. Earlier disease recognition in the community may explain the declining early mortality rate found in our study.

Understanding the differing evolution of survival amid patient subgroups can identify those with higher disease burden and guide future research efforts towards areas of greatest value. An analysis of the distribution of survival gains in the AL amyloidosis population is needed. In this study, we detected significant improvements in survival over time for subgroups based on cardiac involvement and receipt of HDM/SCT, but not for patients with age at diagnosis ≥70 years. Older individuals with AL amyloidosis face distinct clinical challenges [41, 42]. Adverse effects from multiorgan dysfunction in the presence of comorbidities, coupled with poor tolerance of therapies, are magnified in this subgroup. In the latest era of our study, older patients had the highest rate of early mortality. Future efforts for earlier disease recognition are needed for this subgroup. Overcoming these challenges is increasingly important as the median age at diagnosis of AL amyloidosis rises and as more patients survive into older age.

Amongst subgroups based on cardiac involvement and HDM/SCT receipt, survival gains were unevenly distributed. Individuals with absence of cardiac involvement had the greatest improvement with median OS increasing >6 years over the study period. The steepest increase occurred between eras 1 and 2, likely due to the introduction of HDM/SCT. Comparatively, patients with presence of cardiac involvement attained smaller gains at <2 years in total. For those with cardiac stage I/II disease, there was an apparent but non-significantly higher early mortality rate in the modern era (post-2010 vs. pre-2010), thought to be due to statistical probability and bias from censorship of patients lost to follow-up. Meanwhile, for those with cardiac stage III/IIIb disease who are generally ineligible for aggressive treatment with HDM/SCT, survival was found not to have improved with the arrival of bortezomib-based regimens in 2010. Thus, more effective management strategies for patients with advanced amyloid cardiomyopathy are still needed.

HDM/SCT-treated individuals had the highest 5-year OS rate in the latest era, reflecting the potential long-term benefits of this treatment in AL amyloidosis with respect to durable hematologic and organ responses [6, 26, 27, 43]. The >3 year improvement in median OS over time observed in this subgroup may be explained by several factors: (1) improved experience with HDM/SCT in AL amyloidosis, (2) more stringent selection of favorable-risk patients as alternative therapies became available [44], and (3) the ability of HDM/SCT-eligible patients to tolerate sequential treatments as over one-half received ≥2 lines of therapy.

Few studies to date have focused on primary causes of death in AL amyloidosis and how these may change over the course of disease. Available knowledge relies primarily on autopsy studies from the 1980s, in which about 40% of deaths were attributed to progression of cardiac dysfunction or arrhythmias [1, 28]. Autopsy data may not accurately reflect the broader population as cases with more apparent causes of death are less likely to undergo postmortem examination. Our study provides a contemporary report of primary causes of death in a large patient cohort. The most frequent causes were cardiac failure and sudden unexpected death, which together comprised 55% of deaths. Nearly 1 in 3 deaths occurring within 6 months of diagnosis were due to sudden deaths, of which the underlying mechanisms in AL amyloidosis are believed to be electromechanical dissociation (i.e., pulseless electrical activity), unrecognized conduction system disease, thromboembolic events, or abrupt dysautonomia [3, 29, 45–49].

We noted shifts in primary causes of death across disease course. Cardiac failure and sudden death together accounted for 36% of late-occurring deaths, compared to 67% of early-occurring deaths. Meanwhile, renal failure emerged as an important cause of late-occurring deaths. Among long-term survivors, more deaths were also associated with environmental and patient risk factors, such as infections and solid tumor malignancies, indicating potential lasting effects of this disease and its therapies on the host. With longer prospective analysis, these non-cardiac causes of death may be found to be even more prominent.

The majority of deaths captured in our study were directly related to AL amyloidosis. Even among long-term survivors, progression of amyloid organ dysfunction was the top cause. Novel therapies targeting amyloid deposits in tissues, rather than the production of precursor light chains by clonal plasma cells, may enhance organ recovery for these patients. Several anti-amyloid monoclonal antibodies are presently under investigation (e.g., NEOD001 and CAEL-101) [50, 51]. Moreover, advanced tools for disease surveillance, such as minimal residual disease (MRD) assessment, may better prognosticate the risk of late-occurring disease-related events among long-term survivors. One study found that nearly 40% of patients with a durable hematologic complete response over 5 years from last treatment still harbored a detectable clonal plasma cell population by MRD testing [52]. Despite the high proportion of AL amyloidosis-related deaths in our study, there was a significant decline over time. In the latest era, 16% of deaths were from disease-unrelated causes, compared to 3% in the first era. More disease-unrelated deaths are likely to be captured with longer prospective observation. This has the potential to create competing risks in conventional survival analysis, leading to an overestimation of disease risk. Adopting cause-specific frameworks for survival assessment of AL amyloidosis may be beneficial in the future.

Limitations of our study include its single-center, non-prospective design and lack of complete data for all patients. Long-term survival was underrepresented in the later eras due to shorter duration of follow-up. Similarly, this study did not collect robust data on causes of death among long-term survivors, a group that is at greater risk for AL amyloidosis-unrelated causes, because many were alive at data cutoff. Cases with unidentified primary cause of death were also more likely to occur later in disease course and be deemed AL amyloidosis-unrelated. Thus, disease-unrelated mortality is believed to be underestimated in our analysis. Referral center bias may account for higher utilization of HDM/SCT in our study compared to other reports, particularly during era 2 when there was a lack of alternative non-SCT therapies. Median OS may be overestimated and early mortality underestimated as referral bias may enrich our cohort for healthier individuals who have superior survival. We were unable to control for changing referral patterns over time, particularly with regard to cardiac stage as biomarkers were unavailable before 2007. Despite these limitations, this study was strengthened by its large cohort and portrayal of a real-world perspective on survival in AL amyloidosis.

## Conclusions

In order to support future drug development in rare diseases like AL amyloidosis, the U.S. Food and Drug Administration issued a framework in March 2019 for the conduct of natural history studies [53]. By providing insights into disease outcomes in real-world practice, these studies help to define appropriate endpoints and observation periods in clinical trials, and can serve as external comparator arms. The present study provides natural history information on survival and mortality in a large cohort of patients with AL amyloidosis, redefining median OS in the modern era to be 4.6 years. To sustain this progress, ongoing strategies for early disease detection, collaborative clinical trials, along with equitable delivery of care are essential.
